# *Acinetobacter baumannii* Ventilator-Associated Pneumonia: Clinical Efficacy of Combined Antimicrobial Therapy and *in vitro* Drug Sensitivity Test Results

**DOI:** 10.3389/fphar.2019.00092

**Published:** 2019-02-13

**Authors:** Yuqin Huang, Quan Zhou, Wenguo Wang, Qiang Huang, Juan Liao, Junyi Li, Lei Long, Tao Ju, Quan Zhang, Hanqin Wang, Huaqiang Xu, Mingli Tu

**Affiliations:** ^1^Intensive Care Unit, Suizhou Central Hospital, Hubei University of Medicine, Suizhou, China; ^2^Suixian People’s Hospital, Suizhou, China; ^3^Department of Gastroenterology, Tongji Hospital, Tongji Medical College, Huazhong University of Science and Technology, Wuhan, China; ^4^Center for Translational Medicine, Suizhou Central Hospital, Hubei University of Medicine, Suizhou, China; ^5^Department of Respiratory Medicine, Suizhou Central Hospital, Hubei University of Medicine, Suizhou, China

**Keywords:** ventilator-associated pneumonia, *Acinetobacter baumannii*, combined antimicrobial therapy, *in vitro* drug sensitivity test, multidrug-resistant

## Abstract

**Objective:** To evaluate therapeutic efficacy of different combined antimicrobial treatments against *Acinetobacter baumannii* ventilator-associated pneumonia (VAP).

**Methods:** Clinical outcomes were retrospectively analyzed to elucidate the efficacy of four combined antimicrobial regimens. The chessboard and micro broth dilution methods determined the minimum inhibitory concentrations (MICs) of four antiseptic drugs singly used and combined two drugs against 36 isolates of multidrug-resistant (MDR) *A. baumannii*.

**Results:** The incidence of VAP was approximately 6.9% (237/3424) between January 1, 2015 and December 31, and 35.9% (85/237) of the cases were caused by *A. baumannii*. Among these cases, 60 belonged to AB-VAP, for whom antimicrobial treatment plan was centralized and clinical data was complete. Moreover, all 60 strains of *A. baumannii* were MDR bacteria from reports microbiological laboratory. Resistance rate was lowest for amikacin (68.3%) and ampicillin sulbactam (71.7%). Resistance rate for imipenem increased from 63.2 to 90.9% during the 3 years. However, in these 60 cases of AB-VAP, the combination between 4 antibiotics was effective in most cases: the effective rate was 75% (18/24) for sulbactam combined with etilmicin, 71.4% (10/14) for sulbactam combined with levofloxacin, 72.7% (8/11) for meropenem combined with etilmicin, and 63.6% (7/11) for meropenem combined with levofloxacin. There was no statistical difference between four regimens (*P* > 0.05). Sulbactam combined with etilmicin decreased 1/2 of MIC_50_ and MIC_90_ of sulbactam while the decreases in etilmicin were more obviously than single drug. When adopting meropenem combined with levofloxacin or etilmicin, the MIC of meropenem reduced to 1/2 of that in applying single drug. As for sulbactam or meropenem combined with levofloxacin, it also lessened the MIC_50_ of levofloxacin to 1/2 of that for single drug. FIC results suggested that the effects of four combined antimicrobial regimens were additive or unrelated. When sulbactam was combined with etimicin, the additive effect was 63.89%.

**Conclusion:** Drug combination sensitivity test *in vitro* may be helpful for choosing antimicrobial treatment plans. Sulbactam or meropenem as the basis of treatment regimens can function as the alternatives against AB-VAP. Sulbactam combined with etimicin has been regarded as a recommended regimen in Suizhou, Hubei, China.

## Introduction

Ventilator-associated pneumonia (VAP) is a frequent nosocomial infection among critically ill patients (Bouadma et al., 2012). Several clinical studies demonstrated the incidence of VAP is approximately 10% of all mechanically ventilated (MV) patients (Metersky et al., 2005; Wang et al., 2005), with 13.1 VAPs per 1,000 MV-days reported by the International Nosocomial Infection Control Consortium (INICC) during 2010–2015 (Rosenthal et al., 2010). These infections are associated with serious complications, prolonged hospitalization and length of mechanical ventilation, health-care costs, high mortality rate, and infection with multidrug-resistant (MDR) pathogens as well (Muscedere et al., 2010; Kollef et al., 2012; Esperatti et al., 2013).

The isolation of one MDR pathogen has been identified as an independent predictor for increased mortality (Vardakas et al., 2013). Among various gram-negative isolates, the most commonly described MDR pathogens refer to *Acinetobacter baumannii*, *Pseudomonas aeruginosa* and enterobacteriaceae, while MDR-*Acinetobacter baumannii* (MDR-AB) infections mostly consists of VAP (American Thoracic Society and Infectious Diseases Society of America, 2005; Awad et al., 2017). During recent decades, *A. baumannii*, a microorganism characterized by rapid development of resistance to the majority of antimicrobials, has been associated with extremely high mortality rate. Recent research indicated that the incidence of MDR-AB transmission was 315.4 cases/1000 ICU patient-days, and that the mortality rate of patients with MDR-AB ranged from 52 to 66% (Kanafani et al., 2018). Existing data reveal an important disparity in bacterial ecology between countries, and VAP management should be tailored on the basis of local microbiological data. *A. baumannii* is known to be endemic in Asian and European countries (Ayraud-Thévenot et al., 2012; Kanafani et al., 2018). However, data on Chinese are rare, so the aim of this study was to describe epidemiological and clinical characteristics of *A. baumannii* VAP (AB-VAP), and to identify the trend for drugs resisting to antibiotics.

MDR-AB infections are associated with high mortality because of not only affected patients’ critical states, but also the difficulty in treatment (Bassetti et al., 2018). In many ICUs, MDR gram-negative pathogens with limited therapeutic options such as MDR-AB are commonly isolated (Bassetti et al., 2016). Increased incidence of MDR-AB triggers scholars enthusiasm in searching for new treatment options. For VAP patients caused by *A. baumannii*, the use of polymyxins (colistin or polymyxin B) or tigecycline is not recommended according to the 2016 guidelines of the American Thoracic Society and Infectious Diseases Society of America (ATS-IDSA) (Kalil et al., 2016). Colistin application shows an upward tendency due to the emergence of MDR bacterial infections and VAP overseas (Tsioutis et al., 2016). However, none of polymyxins or tigecycline has been widely approved for clinical use in China. In fact, using β-lactamase inhibitor combinations (cefoperazone/sulbactam, ampicillin/sulbactam) or meropenem as the basis of treatment programs accompanied by etilmicin or levofloxacin is frequently applied in empiric antibiotic therapies, but this approach has its defects of drug resistance and drug deficiency for *A. baumannii* in our ICU. The purpose of our study was to elucidate the effects of these empiric antibiotic regimens, and to provide experiential and clinical data for choosing medication regimens. According to the result for drug sensitivity, most VAP cases caused by *A. baumannii* belong to the group of MDR bacteria, so clinical treatment in our ICU mainly adopts combined medication.

In recent years, broad-spectrum antibiotics have been widely used in clinical practice, while the resistance rate of *A. baumannii* exhibits obvious increases (Neonakis et al., 2011; Ayraud-Thévenot et al., 2012). In clinic, the rates of isolating MDR or even extensively resistant *A. baumannii* are increased significantly (Garnacho-Montero and Amaya-Villar, 2010). Studies have shown that the resistance rate of *A. baumannii* to most tested drugs is over 50% (Zhou et al., 2011; Kaliterna and Goic-Barisic, 2013). Therefore, the combination of two or more drugs is often employed in treating MDR-AB infections. However, the sensitivity of drug combination has not been investigated in clinical practice, lacking experimental evidence about drug sensitivity to support the application of combining two or more antibiotics. In this study, 36 strains of MDR-AB were isolated from our ICU in 2017. Based on clinical medication principles, combined antimicrobial susceptibility tests were carried out *in vitro* to explore bacteriostatic effect of our empiric antibiotic regimens, and to provide certain evidence for clinical application. The 3-years retrospective study was conducted to describe the characteristics of AB-VAP, to determine clinical efficacy of four antimicrobials regimens and to study *in vitro* combined antimicrobial susceptibility tests.

## Materials and Methods

### Setting and Study Design

Our retrospective study was conducted in the general ICU of Suizhou Central Hospital Affiliated to Hubei University of Medicine from January 1, 2015 to December 31, 2017. Suizhou Central Hospital is a 1500-bed tertiary care comprehensive hospital, and receives about 59,500 admissions per year. The ICU of the hospital has 26 beds and covers all groups of medical and surgical cases. This study was approved by the Ethics Committee of Suizhou Central Hospital. The requirement for informed consent was waived considering its retrospective design.

The study included all adult patients who were MV for more than 48 h and developed VAP caused by *A. baumannii*. The first episode of AB-VAP or polymicrobial VAP was recorded for each patient. Patients with other previous or concurrent infections were included in the study. Eligible patients were collected on the basis of clinical culture results for the identification of MDR isolates.

### Definitions

Ventilator-associated pneumonia definition conforms to the guidelines of Chinese Thoracic Society (CTS) and the ATS-IDSA (Neonakis et al., 2011; Awad et al., 2017). VAP would be confirmed if radiograph exhibited a new or persistent pulmonary infiltrate, and two or more of the following criteria were met: temperature more than 38°C or lower than 36°C, leukocytosis (peripheral blood leucocyte count >10 × 10^9^/L) or leukopenia (peripheral blood leucocyte count <4 × 10^9^/L) and the presence of purulent bronchial secretions. Pneumonia was considered to be ventilator-associated when the onset occurred 48 h after the initiation of MV, rather than before. Patients with no clinical symptoms or radiological evidence for infiltrate were excluded from the study. VAP onset date was defined as the day when the first clinical positive microbial cultures of aspirate were collected: 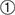
specimen cultures obtained by endotracheal aspiration cultures (ETA) >10^5^ CFU/mL; or bronchoalveolar lavage cultures (BAL) >10^4^ CFU/mL. MDR pathogens commonly resist to at least three classes of the following five antibiotics: cephalosporins, carbapenems, compound preparation containing β-lactamase inhibitor, fluoroquinolone, and aminoglycoside antibiotics. CPIS score is a retrospective calculation value for the studied cases.

### Empirical Antimicrobial Agent Plan and Curative Effect Judgment

Dosage: sulbactam: following routine dose of 1.5 g every 8 h; meropenem: 1 g every 8 h; etilmicin: 100 mg every 12 h; and levofloxacin: 0.6 g daily. Four experiential treatment schemes were as follows: sulbactam + etilmicin, sulbactam + levofloxacin, meropenem + etilmicin, and meropenem + levofloxacin. All drug combination treatments lasted for at least 7–10 days. All patients in the study received appropriate antibiotic therapy.

Clinical outcome of AB-VAP came from comprehensive judgment based on clinical symptoms and clinical pulmonary infection scores (CPIS) of the patients. Cured: clinical symptoms were eliminated and sputum culture reached negative result; Improved: clinical symptoms were obviously relieved and CPIS score was declined when compared to that before combination therapy; Aggravated: clinical symptoms were worse and CPIS score was higher than that before combination therapy; Dead: VAP-related death was defined as that occurring during the treatment when pneumonia signs remained, or that due to septic shock. Effective treatment case number = cured + improved cases, Ineffective treatment cases = aggravated + dead cases.

### Clinical Data Collection

Information about AB-VAP episodes and treatment regimens which adopted β-lactamase inhibitor combinations (cefoperazone/sulbactam, ampicillin/sulbactam) or meropenem as the basis and combined with etilmicin or levofloxacin was collected, and clinical outcomes were analyzed to elucidate the effects of these empiric antibiotic regimens.

Clinical, biological, and treatment data were obtained through retrospectively revising patients’ medical records and nosocomial infection management databases. Clinical data included age, sex, Acute Physiology and Chronic Health Evaluation (APACHE) II scores, ICU admission diagnosis, comorbidities, duration between MV application and VAP onset, and possible risk factors for MDR. Drug sensitivity data for *A. baumannii* to 16 antibiotics between the year of 2015 and 2017 were collected for analyzing drug resistance rate and trend. Data on antimicrobial therapy for AB-VAP group were recorded to assess the effectiveness.

### Sensitivity Test on Drug Combination *in vitro*

Thirty six strains of MDR-AB were isolated from different patients in ICU of our hospital in 2017. Quality control strains were *Escherichia coli* ATCC25922 and *P. aeruginosa* ATCC27853. MH broth functioned as the culture medium.

Minimum inhibitory concentration (MIC) of single drug was determined in accordance with the method recommended by CLSI (M100-S23) (CLSI, 2013). MIC values of sulbactam, meropenem, etilmicin, and levofloxacin against 36 strains of *A. baumannii* were determined using microbroth dilution method. Mueller-Hinton (MH) broth was diluted to a series of concentrations by double ratio. And all of the three antibiotics were diluted to 10 concentration gradients. The concentrations in sensitivity test on drug combination were 512, 256, 128, 64, 32, 16, 8, 4, 2, 1 (μg/mL). According to chessboard method, two different antimicrobial agents in combination with different concentrations and bacterial suspensory were add into 96-hole plates at 37°C for one night. Single drug MIC (MIC_A alone_ and MIC_B__alone_) and MIC value of the optimal combination effect (MIC_A__combined_ and MIC_B__combined_) were recorded. Sensitivity test on drug combination usually adopts fractional inhibitory concentration (FIC) value to evaluate the effect of the combination. Calculating method and interpretation criterion for FIC index were as follows: FIC index = MIC_A__combined_/MIC_A alone_ + MIC_B__combined_/MIC_B alone_, synergistic: FIC≤0.5, addictive: 0.5<FIC≤1, indifference: 1<FIC≤2, and antagonistic: FIC>2.

### Statistical Analysis

SPSS 24.0 and Excel were used for statistical analysis, and *P* < 0.05 meant statistically significant difference. Qualitative variables were expressed as percentages, while quantitative variables as means ± standard deviations (SD) or medians.

## Results

Between January 2015 and December 2017, 3778 adult patients were admitted to our ICU and 3424 of them were MV. Among them, 237 (6.9%, 237/3424) conformed to diagnostic criteria for VAP. Besides, 85 episodes of VAP were attributed to *A. baumannii*, and the incidence of AB-VAP was approximately 35.9% (85/237). Of these 85 patients, 60 were included in our study while 25 others were excluded because VAP treatment time was not long enough or they underwent other treatments. And we analyzed clinical outcome of four different regimens of antibiotics combination: Meropenem or sulbactam with levofloxacin or etilmicin for these 60 cases. Additionally, we also detected the sensitivity for these four regimens based on 36 strains of MDR-AB from different patients in ICU of our hospital in 2017.

### Clinical Characteristics of 60 Patients Treated for AB-VAP

The mean age of the patients was 54.9 ± 12.8 years old (ranging from 29 to 83 years). The ratio of male to female was 4.5 (49 males and 11 females). APACHE II score was 24 ± 5. Among our enrolled patients, 24 (40%) were admitted owing to multiple trauma, 23 (38.3%) because of severe craniocerebral trauma, and 14 (23.3%) for severe nervous system disease. Fourteen patients (22.3%) had history of hypertension, seven (11.7%) had history of chronic obstructive pulmonary disease (COPD), six (10%) had history of other respiratory diseases, 4 (6.7%) had diabetes mellitus, 3 (5%) suffered coronary heart disease, and 3 (5%) endured digestive system disease. The mean duration of hospitalization for VAP was 7.4 ± 5.5 days, while mean span of ICU admission for VAP was 6.9 ± 4.8 days. Besides, the mean period from mechanical ventilation to VAP was 6.4 ± 4.8 days. The duration of mechanical ventilation was 14.6 ± 13.0 days, the duration for ICU stay was 22.7 ± 16.1 days, and that for hospital stay was 34.2 ± 18.4 days. Of the patients, 23 (33.3%) were treated with immunosuppressive therapy for more than 5 days, 14 (23.3%) with immunosuppressive therapy for 3–5 days, and 26 received no immunosuppressive therapy. Clinical characteristics and outcomes of all patients are summarized in Table 1.

**Table 1:** Clinical characteristics of patients treated for *Acinetobacter baumannii* ventilator-associated pneumonia.

Characteristics	
No.	60
Age, mean ± SD (years)	54.9 ± 12.8
Female sex [n (%)]	11 (18.3)
Male sex [n (%)]	49 (81.7)
APACHE II score, mean ± SD	24 ± 5
**ICU admission diagnosis [n (%)]**	
Multiple trauma	24 (40)
Severe craniocerebral trauma	23 (38.3)
Respiratory failure	17 (28.3)
Severe nervous system disease	14 (23.3)
Hemorrhagic shock	7 (11.7)
Various kinds of poisoning	6 (10)
Acute exacerbation of COPD	4 (6.7)
Other reasons	5 (8.3)
**Comorbidities [n (%)]**	
Hypertension	14 (22.3)
COPD	7 (11.7)
Other respiratory diseases	6 (10)
Diabetes	4 (6.7)
Coronary heart disease	3 (5)
Digestive system disease	3 (5)
None	30 (50)
Days hospital admission to VAP, mean ± SD	7.4 ± 5.5
Days ICU admission to VAP, mean ± SD	6.9 ± 4.8
**Days MV to VAP, mean ± SD**	6.4 ± 4.8
Early-onset VAP [n (%)]	7 (11.7)
Late-onset VAP [n (%)]	53 (88.3)
Duration of MV, days, mean ± SD	14.6 ± 13.0
Length of ICU stay, days, mean ± SD	22.7 ± 16.1
Length of hospital stay, days, mean ± SD	34.2 ± 18.4
**Receive immunosuppressive therapy [n (%)]**	
>5 days	20 (33.3)
3–5 days	14 (23.3)
None	26 (43.4)

*APACHE, Acute Physiology and Chronic Health Evaluation; ICU, intensive care unit; COPD, chronic obstructive pulmonary disease; MV, mechanical ventilation*.

### Antimicrobial Resistance of *Acinetobacter baumannii* of VAP From 2015 to 2017

Drug sensitivity of 60 strains of *A. baumannii* to 16 antibiotics between the year of 2015 and 2017 is shown in Table 2.

**Table 2:** Drug sensitivity results on 60 isolates of *Acinetobacter baumannii* to 16 antibiotics between the year of 2015 and 2017 (%).

Antibiotic	2015 (19)	2016 (19)	2017 (22)	All (60)
	
	*R* (%)	*R* (%)	*R* (%)	*R* (%)
Ceftazidime	73.7	78.9	95.5	83.3
Ciprofloxacin	68.4	78.9	95.5	81.7
Gentamicin	68.4	78.9	95.5	81.7
Ceftriaxone	73.7	84.2	95.5	85
Furazolidin	100	94.7	100	98.3
Cefepime	73.7	78.9	90.9	81.7
Imipenem	63.2	78.9	90.9	78.3
Levofloxacin	63.2	73.7	86.4	75
Ampicillin sulbactam	57.9	73.7	81.8	71.7
Compound sulfamethoxazole	73.7	78.9	86.4	80
Tobramycin	78.9	84.2	95.5	86.7
Amikacin	63.2	68.4	72.7	68.3
Piperacillin tazobactam	73.7	78.9	81.8	78.3
Aztreonam	100	94.7	100	98.3
Cefotetan	100	100	100	100
Ampicillin	100	100	100	100

*S, sensitive; R, resistance*.

**Figure 1:**
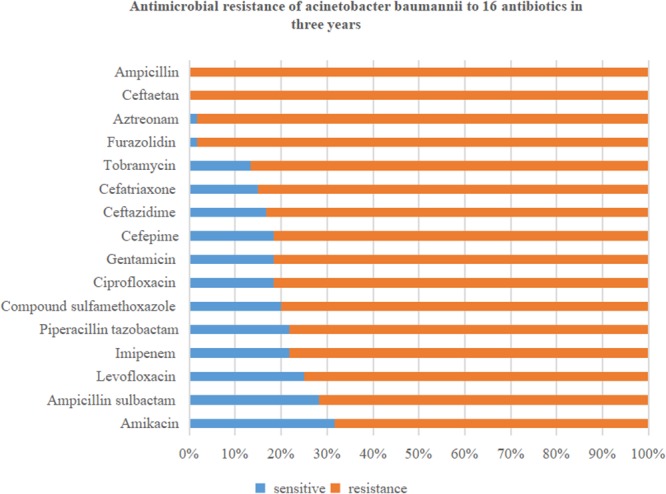
Drug sensitivity of *Acinetobacter baumannii* to 16 antibiotics in 3 years (*n* = 60).

In this retrospective study, 60 strains of *A. baumannii* were all MDR bacteria. Resistance rates to the 16 antibiotics were all over 50%. Specifically, the resistance rate of *A. baumannii* to cefotetan (100%) and ampicillin (100%) was highest, followed by that to furazolidin (98.3%), aztreonam (98.3%), tobramycin (86.7%), ceftriaxone (85%), and ceftazidime (83.3%). The resistance rates to amikacin (68.3%) and ampicillin sulbactam (71.7%) were lowest, followed by that to levofloxacin (75%), imipenem (78.3%), and piperacillin tazobactam (78.3%). The details are shown in Figure 1.

**Figure 2:**
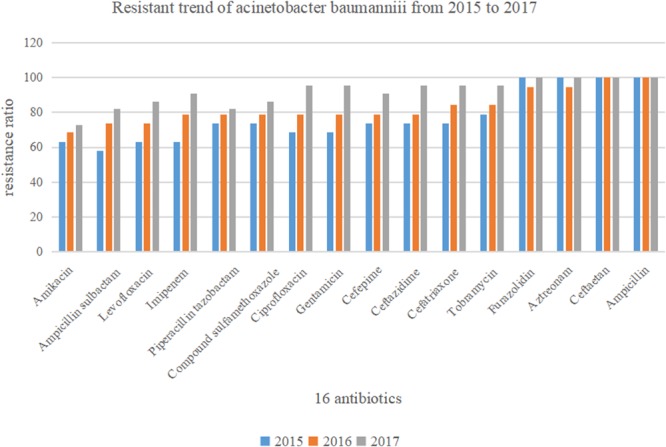
Drug sensitivity of *Acinetobacter baumannii* to 16 antibiotics between the year of 2015 and 2017 (*n* = 60).

Figure 2 shows that drug resistance rate of *A. baumannii* was much higher, with rising tends. Among them, the resistance rate to imipenem, ciprofloxacin, gentamicin, ampicillin sulbactam, and levofloxacin increased rapidly. In particular, the resistance to imipenem increased from 63.2 to 90.9%. While drug resistance to amikacin and piperacillin tazobactam grew relatively slowly.

### Therapeutic Effects of Different Combination Regimens for Four Antibiotics

**Figure 3:**
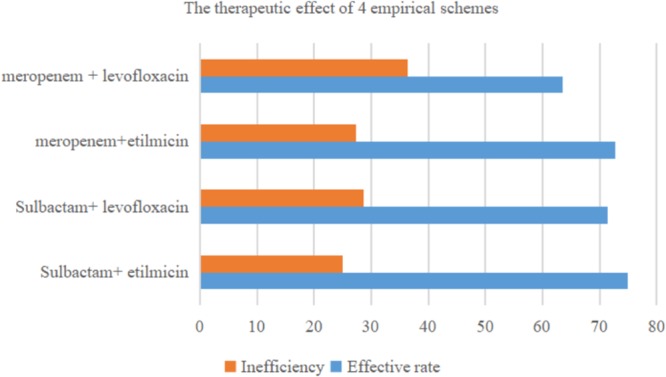
Therapeutic efficacy of four commonly used antimicrobial combinations for *Acinetobacter baumannii* ventilator-associated pneumonia (*n* = 60).

Table 3 shows therapeutic effect of four empirical schemes based on sulbactam or meropenem. In 60 cases, 38 adopted sulbactam as the basis, and 22 received meropenem. In this retrospective study, the most commonly used antibiotic therapy for AB-VAP was sulbactam combined with etilmicin (40% 24/60). Among these cases, sulbactam combined with etilmicin were effective in 18 (75%) and ineffective in 6 (25%). The adoption rate for sulbactam combined with levofloxacin, meropenem with etilmicin, and meropenem with levofloxacin was 23.3, 18.3, and 18.3%, respectively. The effective rate of sulbactam combined with levofloxacin was 71.4%, with an ineffective rate of 28.6%. The effective rate of meropenem combined with etilmicin was 72.7%, and the ineffective rate was 27.3%. The effective and ineffective rates of meropenem combined with levofloxacin was 63.6 and 36.4%, respectively. Therefore, sulbactam combined with etilmicin exhibited best effect. However, there was no statistical difference in therapeutic effect between sulbactam or meropenem. The details are shown in Figure 3.

**Table 3:** Therapeutic effects of four empirical schemes.

Antibiotic therapy (n)	Effective rate (%, n)	*P*-value
Sulbactam + etilmicin (24)	75 (18/24)	0.809
Sulbactam + levofloxacin (14)	71.4 (10/14)	
Meropenem +etilmicin (11)	72.7 (8/11)	0.647
Meropenem + levofloxacin (11)	63.6 (7/11)	
Sulbactam + etilmicin/levofloxacin (38)	73.7 (28/38)	0.649
Meropenem + etilmicin/levofloxacin (22)	68.2 (15/22)	

### Drug Sensitivity for Sulbactam or Meropenem Combined With Etilmicin or Levofloxacin *in vitro*

**Figure 4:**
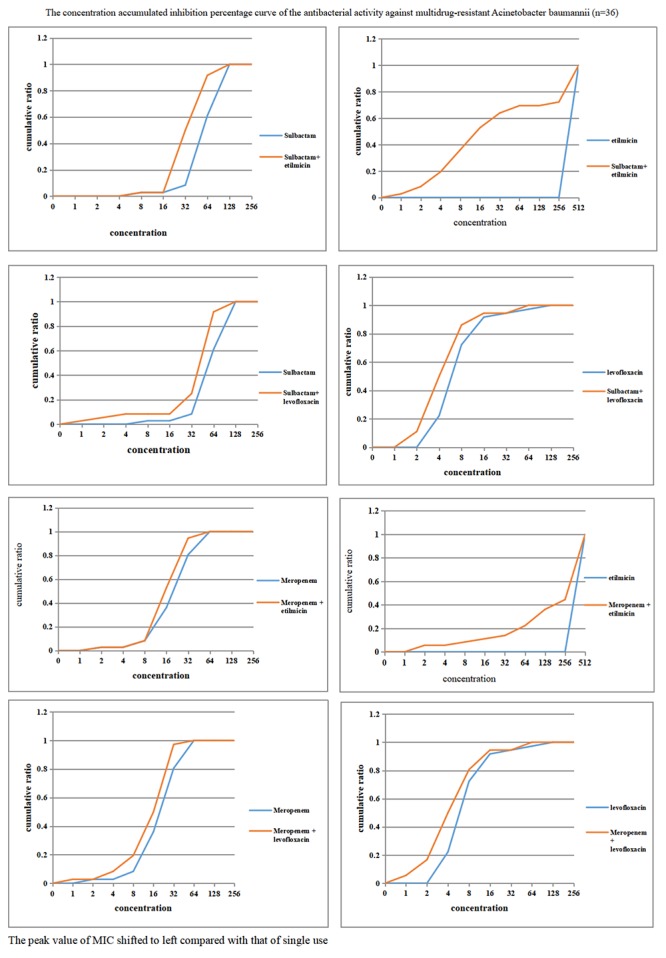
Concentration accumulated inhibition percentage curve of the antibacterial activity against multidrug-resistant *Acinetobacter baumannii* (*n* = 36).

As demonstrated in Figure 4, when sulbactam or meropenem was combined with etilmicin or levofloxacin, respectively, the concentration-cumulative bacteriostatic percentage curve for each drug showed a left shift (better bacteriostatic effect). Table 4 indicates that sulbactam or meropenem led to reduced MIC_50_ and MIC_90_ of the 36 strains, and enhanced antimicrobial activities when combined with etilmicin, especially sulbactam combined with etilmicin. After combined with etilmicin, sulbactam reduced MIC_50_ and MIC_90_ to 1/2. MIC_50_ and MIC_90_ for etilmicin decreased significantly. After combined with levofloxacin or etilmicin, MIC_50_ and MIC_90_ of meropenem were 1/2 of that in single application. After combined with sulbactam or meropenem, the MIC_50_ of levofloxacin was 1/2 of that in single application. The peak value of MIC obviously shifted to the left compared to that in single usage. FIC results in Table 5 suggested that sulbactam or meropenem combined with etilmicin or levofloxacin imposed additive or unrelated effect, without any antagonistic effects. For the combination of sulbactam with etimicin, the synergistic effect was 2.78%, the additive effect was 63.89%, and the unrelated effect was 33.33%; when it came to its combination with levofloxacin, the additive effect accounted for 41.67% while the unrelated effect for 58.33%.

**Table 4:** MICs of four antibiotics against 36 isolates of multidrug-resistant *Acinetobacter baumannii* (μg/mL).

Antibiotics		Alone			Combined		
		MIC_50_	MIC_90_	MIC_G_	MIC_50_	MIC_90_	MIC_G_
Sulbactam + etilmicin	Sulbactam	64	128	8–128	32	64	8–128
	Etilmicin	>512	>512	512	16	512	1–512
Sulbactam + levofloxacin	Sulbactam	64	128	8–128	64	64	1–128
	Levofloxacin	8	16	4–128	4	16	2–64
Meropenem +etilmicin	Meropenem	32	64	2–64	16	32	2–64
	Etilmicin	>512	>512	512	512	512	2–512
Meropenem + levofloxacin	Meropenem	32	64	2–64	16	32	1–64
	Levofloxacin	8	16	4–128	4	16	1–64

**Table 5:** The distribution (%) of FIC for sulbactam or meropenem combined with etilmicin or levofloxacin to multidrug-resistant *Acinetobacter baumannii* strains (*n* = 36).

Antibiotics	FIC≤0.5	0.5<FIC≤1	1<FIC≤2	FIC<2
Sulbactam + etilmicin	2.78 (1)	63.89 (23)	33.33 (12)	0.00 (0)
Sulbactam + levofloxacin	0 (0)	41.67 (15)	58.33 (21)	0.00 (0)
Meropenem +etilmicin	0 (0)	30.56 (11)	69.44 (25)	0.00 (0)
Meropenem + levofloxacin	2.78 (1)	38.89 (14)	58.33 (21)	0.00 (0)

## Discussion

Ventilator-associated pneumonia is a frequent nosocomial infection in critically ill patients and a common complication in MV cases (American Thoracic Society and Infectious Diseases Society of America, 2005). It is the second most common nosocomial infection in the ICU, most common in MV patients (Afshari et al., 2012; Hunter, 2012). VAP is estimated to occur in 9–27% of all MV patients. In our study, the incidence of VAP was approximately 6.9% (237/3424) among all MV patients. VAP is associated with increased hospital stay, health-care costs, mortality, and infection with MDR pathogens (American Thoracic Society and Infectious Diseases Society of America, 2005; Hunter, 2012). VAP could be classified into early-onset and late-onset types, and early-onset VAP (<5d since hospitalization) has been commonly associated with better prognosis, in which bacteria are more susceptible to antibiotic therapy. On the other hand, late-onset VAP refers to VAP occurring 5 or more days after hospital admission, and is associated with higher morbidity, mortality, and MDR pathogens. Of the 60 patients, 7 (11.7%) belonged to early-onset VAP while 53 (88.3%) to late-onset VAP.

Acinetobacter species have become increasingly common in ICUs over the past two decades, causing serious infections. Organism is widely distributed in nature and survives on both moist and dry surfaces (Espinal et al., 2012; Kanafani et al., 2018). Data regarding to VAP caused by *A. baumannii* in China are limited. In this retrospective study, we assessed clinical characteristics and antimicrobial resistance of *A. baumannii* in VAP on the basis of the patients between the year of 2015 and 2017 in our ICU. In our research, the incidence of AB-VAP was approximately 35.9% in all VAP patients. *A. baumannii* has been considered to be a prevailing pathogen causing VAP, leading to high morbidity and mortality among patients in ICUs (Garnacho-Montero et al., 2005; Nhu et al., 2014). *A. baumannii* usually is a MDR bacteria. So AB-VAP patients frequently face high mortality and limited therapeutic options (Bassetti et al., 2018).

In this retrospective study, 60 strains of *A. baumannii* were all MDR bacteria. *A. baumannii* represents a major pathogen of VAP in ICU (El-Saed et al., 2013), and shows high rate of resistance to antimicrobial agents and multiple resistance, which should be noted during clinical process. According to the results on drug sensitivity to 16 commonly used antibiotics, the resistance rate of *A. baumannii* to cefotetan and ampicillin reached 100%, while such figure to furazolidin and aztreonam was over 95%. Comparatively, the resistance rates to amikacin and ampicillin sulbactam were lowest, but still reached 68.3 and 71.7%, respectively. *A. baumannii* resists all antibiotics currently used and such resistance shows an increasing tendency. Among others, its resistance rate to imipenem, ciprofloxacin, gentamicin, ampicillin sulbactam, and levofloxacin increases rapidly. In particular, the resistance to imipenem has increased from 63.2 to 90.9% in 3 years in our study, consistent with the findings from previous studies (D’Arezzo et al., 2011). However, carbapenems (imipenem, meropenem, or doripenem) used to be an excellent choice for serious infections, but the outbreaks of carbapenem-resistant *A. baumannii* infection limit such therapeutic options (Maragakis and Perl, 2008; Chan et al., 2010).

For VAP patients caused by *A. baumannii*, the 2016 guidelines of the ATS-IDSA recommended to adopt polymyxins (colistin or polymyxin B) or tigecycline (Kalil et al., 2016). Colistin application has increased due to the emergence of MDR bacterial infections and VAP overseas (Galani et al., 2008; Tsioutis et al., 2016). Statistical data from meta analysis showed that colistin appeared effective and safe in treating MDR gram-negative bacteria VAP (Gu et al., 2014). Meanwhile, tigecycline is also considered to be one of few therapeutic options for MDR-AB (Kim et al., 2013). However, its role in treating *A. baumannii* bacteraemia remains controversial. Prospective study has demonstrated that tigecycline plus prolonged infusion of standard dose of imipenem/cilastatin exhibits fine clinical effect on patients with MDR-AB VAP (Jean et al., 2016). However, some other studies indicated that tigecycline is associated with higher mortality when compared to control antibiotics (Prasad et al., 2012). Moreover, neither polymyxins nor tigecycline has been widely approved in China due to many aspects, such as economy, drug and hospital management, etc. So large-scale drug resistance monitoring and clinical application data for polymyxins or tigecycline are rare. At present, commonly used antibiotics for *A. baumannii* include β-lactamase inhibitor combinations (cefoperazone/sulbactam, ampicillin/sulbactam), carbapenem antibiotics (meropenem, imipenem), tetracycline antibiotics (minocycline), aminoglycoside antibiotics (etimicin, amikacin), and other quinolones, as well as the third and fourth generations of cephalosporins, which have antibacterial activity targeting *A. baumannii* (Fishbain and Peleg, 2010). In fact, the combination regimens of two or even three drugs are often adopting in treating MDR-AB infections. And the most common combination regimen is based on β-lactamase inhibitor combinations or carbapenem antibiotics. Sulbactam or meropenem as the basis of treatment programs combined with etilmicin or levofloxacin is frequently selected as empiric antibiotic therapy when facing AB-VAP-related drug resistance and drug deficiency in our ICU, though drug resistance was strong and resistance rate of *A. baumannii* to commonly used antibiotics increased rapidly.

In this retrospective study, we analyzed clinical outcome and drug sensitivity for four different combination regimens based on antibiotics: Meropenem or sulbactam with levofloxacin or etilmicin. In 60 AB-VAP cases, antibiotics combination was effective for most cases. Of the cases, 38 received sulbactam as basis, and 22 adopted meropenem as the basis. The most commonly used antibiotic therapy for AB-VAP was sulbactam combined with etilmicin (40% 24/60). The efficiency rate of these four experiential treatment schemes were more than 60%, and the figure for sulbactam combined with etilmicin even reached 75%. A previous study indicated that colistin or ampicillin-sulbactam could reach similar cure rates in treating carbapenem-resistant AB-VAP, but that colistin was associated with higher rates of microbiologic failure, reduction in renal function, and increased 30-day mortality (Zalts et al., 2013). In this study, clinical results indirectly manifested that antibiotics combination against *A. baumannii* (AB) infection in VAP obtained better outcome: According to the result of drug sensitivity in Table 2 and Figure 1, most VAP caused by *A. baumannii* were multi-drug-resistant bacteria, and all 60 strains of *A. baumannii* (in the study) were MDR bacteria from reports microbiological laboratory. However, in these 60 cases of AB-VAP, the combinations between 4 antibiotics were effective in most cases. Therefore, sulbactam or meropenem as the basis of treatment programs combined with etilmicin or levofloxacin may be an efficacious alternative in treating AB-VAP.

To further explore potential mechanisms, drug sensitivity of 36 clinical isolates of MDR-AB were tested *in vitro* to four antiseptic drugs in both single application and drug combination. Sulbactam is a semi-synthetic β-lactamase inhibitor and has direct bacteriostatic effect on *A. baumannii*. Specific *in vitro* activity of sulbactam against Acinetobacter spp. is related to its affinity to penicillin-binding proteins (Pei et al., 2012). Reportedly, the combination of minocycline with cefoperazone-sulbactam exhibited significant synergistic activity against carbapenem-resistant *A. baumannii in vitro* (Pei et al., 2012). *In vitro* drug sensitivity results from Xia et al. (2014) indicated that cefoperazone/sulbactam in combination with minocycline, meropenem and levofloxacin generated synergistic and additive *in vitro* bacteriostatic action on carbapenem-resistant *A. baumannii*. In our study, when sulbactam or meropenem was combined with etilmicin or levofloxacin, respectively, concentration-cumulative bacteriostatic percentage curve for each drug showed a left shift. After combining with etilmicin, MIC_50_ and MIC_90_ of sulbactam decreased 1/2 and MIC_50_ and MIC_90_ of etilmicin also decreased significantly, consistent with conclusion in previous studies (Pei et al., 2012; Xia et al., 2014). Sulbactam or meropenem showed significantly reduced MIC_50_ and MIC_90_, and enhanced antimicrobial activities when combined with etilmicin or levofloxacin. FIC results suggested that the combination of sulbactam or meropenem with etilmicinor levofloxacin mainly produced additive or unrelated effect, without any antagonistic effects. The sensitivity test for drug combination may guide clinical medication. According to *in vitro* sensitivity test, the combination of antibiotics against *A. baumannii* (AB) infection in VAP had higher sensitivity than single antibiotic application: MIC_50_ and MIC_90_ of four antibiotics after the combination were correspondingly decreased compared to those in single antibiotic application. The advantages of combination therapy were more prominent in the combination of sulbactam with etimicin. FIC results suggested the additive effect was 63.89% in such combination regimen.

## Conclusion

Resistance rate of *A. baumannii* to commonly used antibiotics has increased rapidly. *In vitro* sensitivity test on drug combination is helpful choosing antimicrobial treatment regimens. Sulbactam or meropenem as the basis of treatment programs combined with etilmicin or levofloxacin can be an efficacious alternative in treating *A. baumannii* VAP. Sulbactam combined with etimicin is recommended for *A. baumannii* VAP in Suizhou, Hubei, China. Sensitivity test on drug combination may contribute to the formulation of clinical medication plans to some extent.

## Author Contributions

YH and QZ wrote the manuscript, performed the experiments, analyzed the clinical and experimental data, revised the article, and contributed to article design and experimental planning. WW, QH, and JL performed the experiments. JL, LL, TJ, and QZ collected the clinical data. HW, HX, and MT revised the article, and contributed to article design and experimental planning.

## Conflict of Interest Statement

The authors declare that the research was conducted in the absence of any commercial or financial relationships that could be construed as a potential conflict of interest.

## References

[B1] Afshari A. Pagani L. Harbarth S. (2012). Year in review 2011: critical care-infection. *Crit. Care* 16 242–247. 10.1186/cc10425 22152031 PMC3388701

[B2] American Thoracic Society and Infectious Diseases Society of America (2005). Guidelines for the management of adults with hospital-acquired, ventilator-associated, and healthcare-associated pneumonia. *Am. J. Respir. Crit. Care Med.* 171 388–416. 10.1164/rccm.200405-644ST 15699079

[B3] Awad L. S. Abdallah D. I. Mugharbil A. M. Jisr T. H. Droubi N. S. El-Rajab N. A. (2017). An antibiotic stewardship exercise in the ICU: building a treatment algorithm for the management of ventilator-associated pneumonia based on local epidemiology and the 2016 Infectious iseases Society of America/American Thoracic Society guidelines. *Infect. Drug Resist.* 22 17–28. 10.2147/IDR.S145827 29317840 PMC5743123

[B4] Ayraud-Thévenot S. Huart C. Mimoz O. Taouqi M. Laland C. Bousseau A. (2012). Control of multi-drug-resistant *Acinetobacter baumannii* outbreaks in an intensive care unit: feasibility and economic impact of rapid unit closure. *J. Hosp. Infect.* 82 290–292. 10.1016/j.jhin.2012.08.016 23102815

[B5] Bassetti M. Vena A. Castaldo N. Righi E. Peghin M. (2018). New antibiotics for ventilator-associated pneumonia. *Curr. Opin. Infect. Dis.* 31 177–186. 10.1097/QCO.0000000000000438 29337703

[B6] Bassetti M. Welte T. Wunderink R. G. (2016). Treatment of gram-negative pneumonia in the critical care setting: is the beta-lactam antibiotic backbone broken beyond repair? *Crit. Care* 20:19. 10.1186/s13054-016-1197-5 26821535 PMC4731981

[B7] Bouadma L. Wolff M. Lucet J. C. (2012). Ventilator-associated pneumonia and its prevention. *Curr. Opin. Infect. Dis.* 25 395–404. 10.1097/QCO.0b013e328355a835 22744316

[B8] Chan J. D. Graves J. A. Dellit T. H. (2010). Antimicrobial treatment and clinical outcomes of carbapenem-resistant *Acinetobacter baumannii* ventilator-associated Pneumonia. *J. Intensive Care Med.* 25 343–348. 10.1177/0885066610377975 20837632

[B9] CLSI (2013). *Performance Standards for Antimicrobial Susceptibility Testing; Twenty-Third Informational Supplement. CLSI Document M*100-S23. Wayne, PA: Clinical and Laboratory Standards Institute.

[B10] D’Arezzo S. Principe L. Capone A. Petrosillo N. Petrucca A. Visca P. (2011). Changing carbapenemase gene pattern in an epidemic multidrug-resistant *Acinetobacter baumannii* lineage causing multiple outbreaks in central Italy. *J. Antimicrob. Chemother.* 66 54–61. 10.1093/jac/dkq407 21088019 PMC3031335

[B11] El-Saed A. Balkhy H. H. Al-Dorzi H. M. Khan R. Rishu A. H. Arabi Y. M. (2013). Acinetobacter is the most common pathogen associated with late-onset and recurrent ventilator-associated pneumonia in an adult intensive care unit in Saudi Arabia. *Int. J. Infect. Dis.* 17 e696–e701. 10.1016/j.ijid.2013.02.004 23517779

[B12] Espinal P. Marti S. Vila J. (2012). Effect of biofilm formation on the survival of *Acinetobacter baumannii* on dry surfaces. *J. Hosp. Infect.* 80 56–60. 10.1016/j.jhin.2011.08.013 21975219

[B13] Esperatti M. Ferrer M. Giunta V. Ranzani O. T. Saucedo L. M. Li Bassi G. (2013). Validation of predictors of adverse outcomes in hospital-acquired pneumonia in the ICU. *Crit. Care Med.* 41 2151–2161. 10.1097/CCM.0b013e31828a674a 23760154

[B14] Fishbain J. Peleg A. Y. (2010). Treatment of acinetobacter infections. *Clin. Infect. Dis.* 51 79–84. 10.1086/653120 20504234

[B15] Galani I. Flora K. Maria S. Rekatsina P. D. Koratzanis E. Deliolanis J. (2008). Colistin susceptibility testing by Etest and disk diffusion methods. *Int. J. Antimicrob. Agents* 3 434–439. 10.1016/j.ijantimicag.2008.01.011 18328674

[B16] Garnacho-Montero J. Amaya-Villar R. (2010). Multiresistant *Acinetobacter baumannii* infections: epidemiology and management. *Curr. Opin. Infect. Dis.* 23 332–339. 10.1097/QCO.0b013e32833ae38b 20581674

[B17] Garnacho-Montero J. Ortiz-Leyba C. Fernández-Hinojosa E. Aldabó-Pallás T. Cayuela A. Marquez-Vácaro J. A. (2005). *Acinetobacter baumannii* ventilator-associated pneumonia: epidemiological and clinical findings. *Intensive Care Med.* 31 649–655. 10.1007/s00134-005-2598-0 15785929

[B18] Gu W.-J. Wang F. Tang L. Bakker J. Liu J. C. (2014). Colistin for the treatment of ventilator-associated pneumonia caused by multidrug-resistant gram-negative bacteria: a systematic review and meta-analysis. *Int. J. Antimicrob. Agents* 44 477–485. 10.1016/j.ijantimicag.2014.07.004 25199968

[B19] Hunter J. D. (2012). Ventilator associated pneumonia. *BMJ* 344:e3325. 10.1136/bmj.e3325 22645207

[B20] Jean S. S. Hsieh T. C. Hsu C. W. Lee W. S. Bai K. J. Lam C. (2016). Comparison of the clinical efcacy between tigecycline plus extended-infusion imipenem and sulbactam plus imipenem against ventilator-associated pneumonia with pneumonic extensively drug-resistant *Acinetobacter baumannii* bacteremia, and correlation of clinical efcacy with in vitro synergy tests. *J. Microbiol. Immunol. Infect.* 49 924–933. 10.1016/j.jmii.2015.06.009 26341302

[B21] Kalil A. C. Metersky M. L. Klompas M. Muscedere J. Sweeney D. A. Palmer L. B. (2016). Management of adults with hospital-acquired and ventilator-associated Pneumonia: 2016 clinical practice guidelines by the infectious diseases society of america and the american thoracic society. *Clin. Infect. Dis.* 63 e61–e111. 10.1093/cid/ciw353 27418577 PMC4981759

[B22] Kaliterna V. Goic-Barisic I. (2013). The ability of biofilm formation in clinical isolates of *Acinetobacter baumannii* belonging to two different European clones causing outbreaks in the Split University Hospital, Croatia. *J. Chemother.* 25 60–62. 10.1179/1973947812Y.0000000052 23433447

[B23] Kanafani Z. A. Zahreddine N. Tayyar R. Sfeir J. Araj G. F. Matar G. M. (2018). Multi-drug resistant Acinetobacter species: a seven-year experience from a tertiary care center in Lebanon. *Antimicrob. Resist. Infect. Control* 7:9. 10.1186/s13756-017-0297-6 29387343 PMC5778738

[B24] Kim N. H. Hwang J. H. Song K. H. Choe P. G. Kim E. S. Park S. W. (2013). Tigecycline in carbapenem-resistant *Acinetobacter baumannii* bacteraemia: susceptibility and clinical outcome. *Scand. J. Infect. Dis.* 45 315–319. 10.3109/00365548.2012.732705 23113680

[B25] Kollef M. H. Hamilton C. W. Ernst F. R. (2012). Economic impact of ventilator-associated pneumonia in a large matched cohort. *Infect. Control Hosp. Epidemiol.* 33 250–256. 10.1086/664049 22314062

[B26] Maragakis L. L. Perl T. M. (2008). *Acinetobacter baumannii*: epidemiology, antimicrobial resistance, and treatment options. *Clin. Infect. Dis.* 46 1254–1263. 10.1086/529198 18444865

[B27] Metersky M. L. Wang Y. Klompas M. Eckenrode S. Bakullari A. Eldridge N. (2005). Trend in ventilator-associated pneumonia rates between and 2013. *JAMA* 2016 2427–2429. 10.1001/jama.2016.16226 27835709

[B28] Muscedere J. G. Day A. Heyland D. K. (2010). Mortality, attributable mortality, and clinical events as end points for clinical trials of ventilator-associated pneumonia and hospital-acquired pneumonia. *Clin. Infect. Dis.* 51(Suppl. 1), S120–S125. 10.1086/653060 20597661

[B29] Neonakis I. K. Spandidos D. A. Petinaki E. (2011). Confronting muhidrug-resistant Acinetobacter baumanii: a review. *Int. J. Antimierob. Agents* 37 102–109. 10.1016/j.ijantimicag.2010.10.014 21130607

[B30] Nhu N. T. K. Lan N. P. H. Campbell J. I. Parry C. M. Thompson C. Tuyen H. T. (2014). Emergence of carbapenem-resistant *Acinetobacter baumannii* as the major cause of ventilator-associated pneumonia in intensive care unit patients at an infectious disease hospital in Southern Vietnam. *J. Med. Microbiol.* 63 1386–1394. 10.1099/jmm.0.076646-0 25038137 PMC4170484

[B31] Pei G. Mao Y. Sun Y. (2012). In vitro activity of minocycline alone and in combination with cefoperazone-sulbactam against carbapenem-resistant *Acinetobacter baumannii*. *Microb. Drug Resist.* 18 574–577. 10.1089/mdr.2012.0076 22928863

[B32] Prasad P. Sun J. Danner R. L. Natanson C. (2012). Excess deaths associated with tigecycline after approval based on noninferiority trials. *Clin. Infect. Dis.* 54 1699–1709. 10.1093/cid/cis270 22467668 PMC3404716

[B33] Rosenthal V. D. Al-Abdely H. M. El-Kholy A. A. AlKhawaja S. A. Leblebicioglu H. Mehta Y. (2010). International Nosocomial Infection Control Consortium (INICC) report, data summary of 50 countries for -2015: device-associated module. *Am. J. Infect. Control* 2016 1495–1504. 10.1016/j.ajic.2016.08.007 27742143

[B34] Tsioutis C. Kritsotakis E. I. Karageorgos S. A. Stratakou S. Psarologakis C. Kokkini S. (2016). Clinical epidemiology, treatment and prognostic factors of extensively drug-resistant Acinetobacter baumanni ventilator-associated pneumonia in critically ill patients. *Int. J. Antimicrob. Agents* 48 492–497. 10.1016/j.ijantimicag.2016.07.007 27542315

[B35] Vardakas K. Z. Rafailidis P. I. Konstantelias A. A. Falagas M. E. (2013). Predictors of mortality in patients with infections due to multidrug resistant Gram negative bacteria: the study, the patient, the bug or the drug? *J. Infect.* 66 401–414. 10.1016/j.jinf.2012.10.028 23142195

[B36] Wang Y. Eldridge N. Metersky M. L. Verzier N. R. Meehan T. P. Pandolfi M. M. (2005). National trends in patient safety for four common conditions, -2011. *N. Engl. J. Med.* 2014 341–351. 10.1056/NEJMsa1300991 24450892 PMC4042316

[B37] Xia J. Zhang D. Xu Y. Gong M. Zhou Y. Fang X. (2014). A retrospective analysis of carbapenem-resistant *Acinetobacter baumannii*-mediated nosocomial pneumonia and the in vitro therapeutic benefit of cefoperazone/sulbactam. *Int. J. Infect. Dis.* 23 90–93. 10.1016/j.ijid.2014.01.017 24726664

[B38] Zalts R. Neuberger A. Hussein K. Raz-Pasteur A. Geffen Y. Mashiach T. (2013). Treatment of carbapenem-resistant *Acinetobacter baumannii* ventilator-associated pneumonia: retrospective comparison between intravenous colistin and intra-venous ampicillin-sulbactam. *Am. J. Ther.* 23 e78–e85. 10.1097/MJT.0b013e3182a32df3 24263165

[B39] Zhou H. Zhang T. Yu D. Pi B. Yang Q. Zhou J. (2011). Genom ic analysis of the multidrug resistant *Acinetobacter baumanni* strain MDR-ZJ06 widely spread in China. *Antimierob. Agents Chemother.* 55 4506–4512. 10.1128/AAC.01134-10 21788470 PMC3187012

